# Medical Ethnobotany in Europe: From Field Ethnography to a More Culturally Sensitive Evidence-Based CAM?

**DOI:** 10.1155/2012/156846

**Published:** 2012-07-30

**Authors:** Cassandra L. Quave, Manuel Pardo-de-Santayana, Andrea Pieroni

**Affiliations:** ^1^Center for the Study of Human Health, Emory University, 550 Asbury Circle, Candler Library 107, Atlanta, GA 30322, USA; ^2^Departamento de Biología (Botánica), Universidad Autónoma de Madrid, c/Darwin 2, Campus de Cantoblanco, E-28049 Madrid, Spain; ^3^University of Gastronomic Sciences, Piazza Vittorio Emanuele 9, 12060 Bra/Pollenzo, Italy

## Abstract

European folk medicine has a long and vibrant history, enriched with the various documented uses of local and imported plants and plant products that are often unique to specific cultures or environments. In this paper, we consider the medicoethnobotanical field studies conducted in Europe over the past two decades. We contend that these studies represent an important foundation for understanding local small-scale uses of CAM natural products and allow us to assess the potential for expansion of these into the global market. Moreover, we discuss how field studies of this nature can provide useful information to the allopathic medical community as they seek to reconcile existing and emerging CAM therapies with conventional biomedicine. This is of great importance not only for phytopharmacovigilance and managing risk of herb-drug interactions in mainstream patients that use CAM, but also for educating the medical community about ethnomedical systems and practices so that they can better serve growing migrant populations. Across Europe, the general status of this traditional medical knowledge is at risk due to acculturation trends and the urgency to document and conserve this knowledge is evident in the majority of the studies reviewed.

## 1. Introduction

European folk medicine has held a special fascination for ethnographers, anthropologists and ethnobiologists alike. Rooted in a long history of tradition dating back to ancient Greek, Roman, and Arabic medical theories, this folk knowledge has been passed down via both written and oral pathways over the centuries. While some of these medical traditions have survived the passage of time relatively intact, many others have changed or disappeared, while “new” remedies and uses of plants have also emerged. 

Today, European traditional medical knowledge is in a state of flux. In many cases, local traditional knowledge regarding the environment, wild food and medicine sources, and human health is in an alarming state of decline. This has prompted researchers to pursue field studies with the aim of documenting, preserving, and comparing data concerning these unique local ethnomedical practices. On the other hand, the mainstream popularization of certain complementary and alternative remedies for human health has promoted common knowledge of some heavily marketed species (many of which are nonnative to Europe). However, herb-drug interactions regarding these popular products are still poorly understood in most cases and present a dilemma for the European allopathic medical community (e.g., see [1] for a patient case study on self-medication with valerian and passionflower in addition to the prescribed anxiolytic drug, lorazepam). Furthermore, safety concerns resulting from decreased liver function and even hepatotoxicity in patients that self-medicate with herbs (sometimes due to use of the incorrect species) also merit the close attention of the medical community [2, 3]. 

Ethnobiological field studies in Europe can enhance our understanding not only of traditional healthcare practices, but also provide insight into human health and offer new solutions for food security. Specifically, ethnobiological data are useful to medical practitioners charged with the care of migrant and other populations that use CAM in that it can provide a basis for understanding folk medical beliefs about sickness, health, and therapies. Moreover, much research into the medicinal and nutritional value of plants that are presently underused in mainstream culture may actually lead to the development of the foods, pharmaceuticals, and CAMs of tomorrow. 

### 1.1. A Brief History of Medicinal Plant Use in Europe

Europe represents a melting pot of culture and has a long history of transmission of knowledge of medical practices across geographic, cultural, and linguistic borders. Early *Materia Medica* and medical tomes by scholars like the Pedanius Dioscorides [4] and Avicenna (Ibn Sina) [5, 6] heavily influenced early medicine in Europe, resulting in the later production of numerous herbal texts, especially during the middle ages (A.D. 500–1400). Everyday medical needs were met in the household and more critical care was offered through religious outlets, such as monasteries, where herbal physic gardens were used to maintain important medicinal species [7]. The early pharmacopoeia of Europe was based in large part on products of botanical, animal, and mineral origin. Plant materials were collected or grown locally, and more exotic medicines, including spices like black pepper (*Piper nigrum *L., Piperaceae) and nutmeg (*Myristica fragrans *Houtt., Myristicaceae), became accessible through early land and, later, sea trade routes [8, 9]. Today, this tradition of incorporating exotics into the CAM pharmacopoeia continues throughout Europe, and examples of popular nonnative herbal CAM products include those containing arnica (*Arnica montana *L., Asteraceae) [10], cinnamon (*Cinnamomum *spp., Lauraceae) [11], ginseng (*Panax ginseng *C. A. Mey., Araliaceae) [12], and ginkgo (*Ginkgo biloba *L., Ginkgoaceae) [13], among others.

While many of the same plants popular today in European folk medicine have been in use for centuries, if not millennia—the ways in which they are used is often quite different from that documented in historical texts. Furthermore, there is extensive variation in the current day preparation and indication for use of medicinal plants across geographic and cultural planes, and this is clearly supported by the existing ethnobotanical literature concerning Europe. 

## 2. Methodology

**Figure 1:**
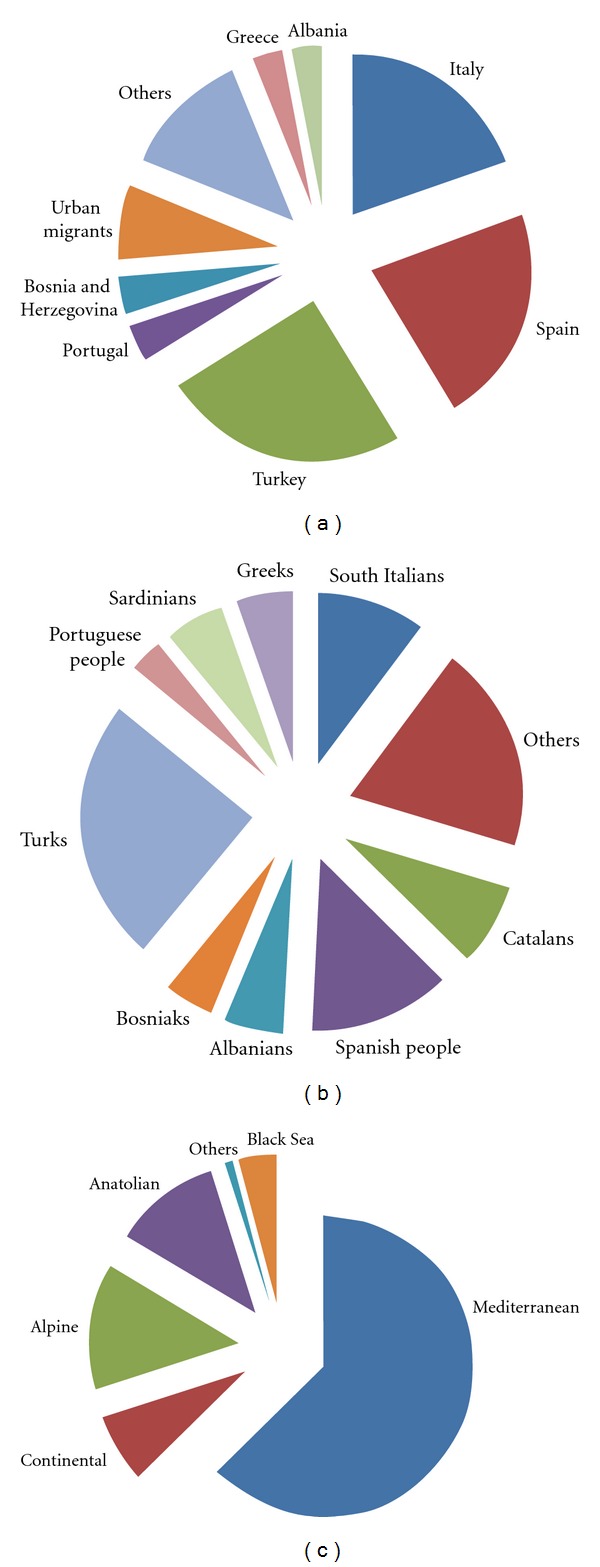
Representation of medicoethnobotanical studies included in our analysis as they relate to the (a) involved countries, (b) ethnic groups and, (c) biogeographical regions.

This review is based on an exhaustive survey of medicoethnobotanical field studies conducted in Europe over the last two decades (1992–2012) that have been indexed by Scopus [14–130]. Our aim was to analyze the relative influence of different European countries, ethnic groups, and bio-geographical regions on the state of current European CAM. Given the importance of other less accessible studies to understanding the relevance of local traditional knowledge to CAM practices, a nonexhaustive list of local publications or PhD theses were also considered in the discussion of case studies of field ethnobotany in SW Europe—the Iberian Peninsula, in particular. These studies, however, were not included in our overall analysis of the data, which is based the Scopus search, reported in Figure 1. 

For the purposes of this review, we have defined Europe to include the European continent plus Cyprus, Turkey, the Caucasus, and the Azores/Madeira/Canary Isles. We did not consider reviews or meta-analyses of preexisting data. Our criteria for the inclusion and exclusion of studies considered are detailed in Table 1. Figure 1 illustrates the data represented as they relate to the involved countries, ethnic groups, and biogeographical regions, respectively. Family assignments for all plants discussed in this review follow Angiosperm Phylogeny Group III guidelines [131, 132].

## 3. Medical Ethnobotany in Europe

Here, we have divided our discussion of medical ethnobotanical field studies in Europe into three general geographic regions: SW Europe, SE Europe, and the rest of Europe. We have placed the most emphasis in our discussion of the European literature on SW Europe in order to provide a detailed discussion with specific case studies and examples of the relevance of traditional knowledge recorded in field studies to future European CAM therapies.

### 3.1. Medical Ethnobotany in SW Europe

The Iberian Peninsula can be considered a small continent of around 600,000 km^2^. It is separated from the rest of Europe by the Pyrenees, a mountainous barrier that has contributed to its relative isolation. It has a striking climatic, geological, geographical, biological, cultural, and linguistic diversity. Its vascular plants flora, with around 7,500 taxa, is one of the richest of Europe [133]. Lusitanians, Basques, Celts, Phoenicians, Greeks, Romans, Vandals, Arabs, and many other ethnicities and cultures have historically populated the region and the Iberian Peninsula is, therefore, considered a crossroad of civilizations. This continuum of migration and displacement since the earliest periods has contributed to a constant exchange of plants, ideas, customs, and knowledge. In this sense, the key role played by Portuguese and Spanish people in introducing American plants and their knowledge in Europe is especially relevant [134]. This rich biocultural diversity has become translated into a very deep ethnobiological knowledge that unfortunately has only partially reached us. Remnants of the wisdom and practices of these cultures can be traced in plant names, home remedies, or gastronomic recipes [135, 136].

Until the 1950s, Iberian society was mainly agrarian and rural. Most people were subsistence farmers and markets were weakly developed. There were exceptions such as some industrial regions in Catalonia or the Basque Country and big cities such as Madrid, Barcelona, Lisbon, or Porto. Many remote places remained isolated and only local markets, livestock fairs, the annual visit of transhumance herders, or an incipient tourism interrupted their isolation [137]. Professional medical care was not accessible for many rural people, since it was too expensive, and they could not afford it and, furthermore, many villages did not have doctors in the vicinity. Self-care prevailed, and most people relied in their knowledge about home-remedies and the wisdom of other members of the community. Local healers, in particular, played a key role in both veterinary and human health care [138].

Deep transformations in the lifestyle of rural societies began in the 1960s with the process of industrialization and mechanization of the farms and the shift from a rural, agriculturally based, subsistence economy to a market oriented one [139]. This process has not been uniform throughout the region and there are some areas that completed the transition only within the past two decades. Millions of people migrated to other countries or to Iberian cities [137, 140]. Most young people preferred to adapt to the new ways of life, and they rejected the wisdom of their ancestors with the subsequent loss of an important part of the traditional ecological knowledge [141]. The National Health System spread until it provided universal coverage at the end of the 1970s in Portugal and the 1980s in Spain [142]. As domestic healthcare has been commonly considered by official medicine as an old practice that should be abolished, medicinal plant use came to be considered as a symbol of poverty or backwardness [138, 143].

Though not as common as in the past, there are still people who remember how life was when they mainly relied on the plants, animal, and materials found in their surroundings for food, medical, and other basic needs. However, the lack of direct contact with nature while tending animals, agricultural fields or home gardens has led to a strong erosion of this traditional ecological knowledge (TEK) and it is essential to record it before it is too late [138].

#### 3.1.1. Research in Iberian Medical Ethnobotany

The rich traditional lore of the Iberian Peninsula has attracted many folklorists, ethnographers, and medical anthropologists and ethnobotanists since the end of the nineteenth century [144–148]. However, systematic ethnobotanical studies substantiated with reliable botanical identification, did not become the general standard until the 1980s [149]. Since then, Ethnobotany—especially Medical Ethnobotany—has grown rapidly in Spain and Portugal. This renewed interest has led to the creation of research groups in many universities and research centers (e.g., Instituto Politécnico de Bragança, Jardín Botánico de Castilla-La Mancha, Jardín Botánico de Córdoba, Museu Botânico de Beja, Universidad Autónoma de Madrid-IMIDRA-Real Jardín Botánico-CSIC, Universidad de Alicante, Universidad de Extremadura, Universidad de Granada, Universidad de Murcia, Universidade de Évora, Universitat de Barcelona). 

More than 30 Ph.D. theses have been fully or partially devoted to the study of medical ethnobotany of Spanish and Portuguese territories (e.g., see [150–157]), and a high number of other surveys have been conducted in the last three decades (e.g., see references in Tables 1.1 and 1.2 in Carvalho 2005 [156], for Portugal and references in Appendix 1 in Morales et al. 2011 [133] for Spain and Table 2 in this paper). This has resulted in the Iberian Peninsula being one of the European regions with the largest number of ethnobotanical studies [141].

Many of these surveys have been published only locally (e.g., see [146, 147, 165, 166]) or are unpublished Ph.D., master, or graduate theses that are not accessible to an international audience (e.g., see [163, 167, 168]). Therefore, in order to facilitate its access, internet repositories have been created both in Portugal (http://www.etnobotanica.uevora.pt/) and Spain (http://bibdigital.rjb.csic.es/spa/index.php). Given the importance of benefit-sharing and returning and facilitating the conservation and dissemination of traditional knowledge to local stakeholders, many books have been written for a general public audience (e.g., see [169–171]). There are also a number of studies that have been published in national or international scientific journals, such as *Journal of Ethnopharmacology* [84, 105, 108], *Economic Botany *[111, 172, 173], *Journal of Ethnobiology and Ethnomedicine* [83, 128, 174], or *Revista de Estudios Extremeños* [175–177].

This rich level of production is reflective of the increasing social, political and scientific interest in traditional knowledge and specifically medical ethnobotany and the need to promote and conserve it. In fact, the Spanish legislation has assumed the principles of the Convention on Biological Diversity (CBD) in the law on Natural Heritage and Biodiversity [178] and in the Royal Decree that regulates the Spanish Inventory of Natural Heritage and Biodiversity [179]. 

#### 3.1.2. Traditional Iberian Pharmacopoeia

According to a recent review of medicinal plants popularly used in Spain [180], the number of species employed is around 1,200, more than 15% of the Iberian flora. The figure of plants used in the Iberian Peninsula is surely remarkably higher, since the review does not include Portuguese or many Spanish studies. However, the richness of species is only a poor indicator of ethnomedical knowledge, since the number of remedies or medicinal plant uses is several times bigger. For instance, in Campoo, the 160 species used actually corresponds to 439 plant uses [160]. Likewise, in Montesinho, 169 medicinal species corresponded to 509 plant uses [159]. Moreover, in addition to the predominant role played by medicinal plants in local pharmacopoeias, it must be noted that many animal- and mineral-based remedies also serve a key role in folk-medical practices [105, 160, 177, 181–183]. 

In Iberia, more than 400 plants were used in the richest area, Pallars, a territory of the Catalan Pyrenees [61, 64]. These figures cannot be easily compared since there are significant differences in the study sites (area, population, richness of the flora) and in sample size (number of localities visited, and of informants interviewed). Medicinal plants were used for humans and animals, with the human pharmacopoeia usually being richer than the ethnoveterinary *materia medica*. For instance, 166 and 32 species were used in human and animal medicine in Montesinho, NE Portugal, 154 and 86 in Campoo, Cantabria [160], or 229 and 60 to the west of the Granada province respectively [81, 105].

Medicinal plants were mainly used for common disorders such as catarrh, pneumonia, fever, diarrhea, stomach and intestinal disorders, high blood pressure, wounds, bruises, or muscular pains. Many surveys concluded that digestive, respiratory, and skin disorders were among those most commonly treated with home remedies [159, 184].

Households commonly kept a few species for treating the most common disorders, serving as a sort of traditional First Aid Kit. Their contribution was essential to the families' well-being [138, 150]. This group of species is specific to each geographic area and included those species with the highest frequency of citation. This knowledge belonged to the collective memory of each area. For instance, in Gorbeialdea (Basque Country), this traditional medical repository contained *Urtica dioica *L. (Urticaceae) and* Verbena officinalis *L. (Verbenaceae) for respiratory disorders, *Chelidonium majus* L. (Papaveraceae) and* Allium cepa *L. (Amaryllidaceae) for skin conditions, *Plantago lanceolata *L. (Plantaginaceae) and other* Plantago species *for musculo-skeletal disorders, *Chamaemelum nobile *(L.) All. (Asteraceae) and *Helleborus viridis *L. (Ranunculaceae) for digestive diseases, and *Urtica dioica *for circulatory conditions [164].

Apart from those plants whose knowledge was shared by most people, there were also plants and remedies known only by specialists, such as healers or local experts with a wider extensive knowledge of herbs. Particular recipes made of plant mixtures, some herbal extracts, and special lotions and ointments were prepared by healers or wise women who provided them on request [138]. Other types of specialized medical therapies, such as the treatment of broken bones and many ethnoveterinary remedies were only applied by local healers [160, 185].

Some of these local experts were incredibly wise and had a precious store of extensive traditional knowledge. For instance, Palacín found in his ethnobotanical survey of Aragon, in which he interviewed more than 1,500 informants, that three women knew more than hundred medicinal plants [186]. One of them knew 230 medicinal plant species, 31 animals and 29 minerals with which she could prepare more than 1,450 remedies, a really extraordinary example of a traditional knowledge keeper. To record such an amount of knowledge was not easy, and Palacín had to interview her 69 times over a period of 6 years. Women have been recognized as having a deeper knowledge of traditional health strategies than men in many studies around the world [160, 177, 187].

Lamiaceae, Asteraceae, and Rosaceae are always among the most important families referred in these territories [35, 62, 81] as happens in many other ethnopharmacopoeias around the world [187–189]. A clear positive selection for the species of these families explains this preference. Here, we use a nonexhaustive list of species as examples to demonstrate a classification system, which is dependent on both the distribution of use and the origin of the plant species.

1. *Common and abundant wild species with a wide distribution area*: this group includes examples such as *Chelidonium majus *L. (Papaveraceae)*, Crataegus monogyna *Jacq. (Rosaceae)*, Chamaemelum nobile *L. (All.) (Asteraceae)*, Foeniculum vulgare *Mill. (Apiaceae)*, Malva sylvestris *L. (Malvaceae)*, Mentha pulegium *L. (Lamiaceae)*, Paronychia argentea *Lam. (Caryophyllaceae)*, Santolina chamaecyparissus *L. (Asteraceae)*, Rosmarinus officinalis *L. (Lamiaceae)*, Sambucus nigra *L. (Adoxaceae), and *Thymus vulgaris *L. (Lamiaceae) [180]. This group includes the most common species, widely used throughout the Peninsula. Most of them are also commonly used in other European countries, including Italy (e.g., see [47, 56, 68]), Greece [59, 67, 125], and Turkey [87, 88], among others.
2. *Species with a broad range, but not abundant and highly appreciated*: this includes *Arnica montana *L. (Asteraceae)*, Sideritis hyssopifolia *L. (Lamiaceae)*, Gentiana lutea *L. (Gentianaceae)or *Osmunda regalis *L. (Osmundaceae), (e.g., [190, 191]).
3. *West European or Iberian endemisms with a widespread use*: this group includes common, widely used, and highly valued species such as *Jasonia glutinosa *DC. (Asteraceae)*, Centaurea ornata *Willd. (Asteraceae)*, Thymus mastichina *L. (Lamiaceae) [192, 193],and other more restricted such as* Lilium pyrenaicum *Gouan (Liliaceae)*, Lithodora fruticosa *(L.) Griseb. (Boraginaceae), and* Phlomis lychnitis* L. (Lamiaceae) [180, 190].
4. *Restricted endemisms*: this includes *Artemisia granatensis *Boiss. (Asteraceae)*, Erodium petraeum *Willd. (Geraniaceae)*, Santolina oblongifolia *Boiss. (Asteraceae)and* Thymus moroderi *Pau ex Martinez (Lamiaceae) [149, 180].
5. *Cultivated species whose use is very popular*: this group includes *Allium cepa *L.*, A. sativum *L. (Amaryllidaceae)*, Citrus limon *(L.) Osbeck (Rutaceae)*, Chenopodium ambrosioides *L. (Amaranthaceae)*, Bidens aurea *(Aiton) Sherff (Asteraceae)*, Hylotelephium telephium *(L.) H. Ohba (Crassulaceae)*, Juglans regia *L. (Juglandaceae)*, Laurus nobilis *L. (Lauraceae)*, Matricaria recutita *L. (Asteraceae)*, Melissa officinalis *L. (Lamiaceae)*, Olea europaea *L. (Oleaceae)*, Ruta chalepensis *L. (Rutaceae)*, Tilia platyphyllos *Scop. (Malvaceae)*, Vitis vinifera* L. (Vitaceae), and *Zea mays *L. (Poaceae) [180].

Despite the fact that many of these plants have been widely used, they are abundant and have not suffered overexploitation. These species have the essential characteristics for being used in elementary healthcare: they are widespread, easily gathered, and have a vast array of medicinal properties and pharmacological effects [138].

On the other hand, there are also species that have suffered overexploitation. For example, in the case of *Artemisia granatensis *Boiss. (Asteraceae), an endangered species endemic to Sierra Nevada, its high demand eventually led to increased scarcity and the threat of extinction. Therefore, it was officially protected in 1982 [194]. This case, however, seems to be the exception more than the rule. For example, in other cases like that of *Osmunda regalis *L. (Osmundaceae), local management practices have helped to make its use sustainable. A study of traditional knowledge and management of this species in Cantabria found that some people were concerned about the rising demand from urban areas, since people from cities were unaware of the ecology of the fern. The scarcity of the fern has led rural residents to develop practices such as keeping its location secret, not harvesting the complete rhizome in order to avoid killing the plant and allowing its regeneration, and cultivating the species in home gardens [195]. The introduction and protection of wild medicinal species in home gardens has been also recorded in many other regions of Spain, Portugal and Austria [138, 192, 196].

#### 3.1.3. Research in Italian Medical Ethnobotany

The Italian peninsula and islands (including Sardinia and Sicily) comprise a land mass of roughly 300,000 km^2^. The vascular flora includes 6,711 species [197], which are distributed across geographic regions of mountains, hills, and plains [198]. Much like the Iberian peninsula, the rich lore and folk medical traditions of Italy attracted the attention of many scholars in the 19th to the first half of the 20th century (e.g., see the works of Giuseppe Ferraro [199, 200], Giovanni Pons, [201, 202], Giuseppe Pitrè [203], Oreste Mattirolo [204], Ernesto de Martino [205], and Caterina Chiovenda-Bensi [206, 207]). However, it has only been in the past forty years or so that more systematic ethnobotanical surveys throughout Italy have emerged (see, e.g., [28, 31, 40, 41, 44–47, 53–56, 60, 65, 68, 98, 104, 126, 208–211]).

#### 3.1.4. Traditional Italian Pharmacopoeia

Like the field studies conducted throughout the Iberian Peninsula, recent ethnobotanical studies undertaken over the past five years in Italy have also revealed a rich traditional pharmacopoeia that utilizes both local flora and fauna. Indeed, a multisite study of the zootherapeutic practices in select rural communities in several countries—including Italy (Basilicata), Spain, and Albania—revealed the use of 21, 11, and 34 animal species used in multiple ways as ingredients in the treatment of 50 (etic) categories of disease or illness [181]. Furthermore, there is also a strong documented tradition of use of plant remedies in the sites where these studies were performed. For example, in one study conducted in Basilicata, which focused only on the topical use of plants for the treatment of skin and soft tissue infection, 116 distinct remedies coming from 38 medicinal plant species were documented [41]. 

In other regions of Italy, traditional knowledge of medicinal plants is also still quite resilient. For example, in Campania, a study examining a broad range of medicinal applications of plants recorded traditional knowledge concerning 95 medicinal species, representing roughly 24% of the entire local flora [46]. In Liguria, a total of 367 distinct use reports concerning 82 medicinal species was recorded along with reports of high levels of dietary intake of wild species—likely serving as functional or medicinal foods [211]. In Molise [40] and Valvestino [31], the medicinal uses of 64 and 58 species were recorded, respectively. A 2011 study of the folk phytotherapy along the Amalfi coast revealed that 102 medicinal plants are used for medicinal purposes, with a total of 276 distinct uses [210]. One of the most interesting findings of this study was that 62% of the recorded uses were still in common practice, supporting the idea that though not necessarily reported to biomedical care providers, many Italians do commonly use CAM therapies as a key mode of therapy. Furthermore, this “hidden” practice of local CAM use is likely prevalent especially throughout southern Europe, where there is still a relative prevalence of traditional knowledge concerning folk therapies.

### 3.2. Medical Ethnobotany in SE Europe

Quite similarly to the examples presented of SW Europe, the SE regions have been subject to political and economic shifts that have heavily influenced local lifeways, economies, foodways, connectivity with nature, and as a consequence, transmission of traditional knowledge regarding health and local CAM practices. The rural regions of SE Europe represent some of the most vibrant scenarios for conducting medical ethnobotanical studies (see, e.g., field studies in Croatia [37, 112], Bosnia and Herzegovina [118], Albania [119, 212], Serbia [52, 213], Kosovo [214], Turkey [215], and Greece [59, 67, 125]). The reasons are numerous.

1. This mountainous area is a hotspot for both biodiversity and cultural/ethnic diversities.
2. The area has historically provided the botanical materials that are sold in the Western European herbal market (especially during the last few centuries).
3. The majority of dried medicinal plants and an impressive number of locally gathered medicinal plants are still widely used in many households for local healthcare.
4. Medicinal plants are central to many economic initiatives and programs devoted to rural development.

Moreover, medical ethnobotany studies in the Western Balkans (e.g., see [19, 26, 27, 59, 67, 112, 114, 118, 121, 216]) provide a unique arena for cross-cultural analysis of local uses of medicinal plants, which can contribute to the identification and development of a better understanding of factors that affect changes in plant uses and perceptions.

The ethnopharmacopeia of SE Europe shares some similarities with that of SW Europe, especially with regards to some of the most common medicinal species, including *Allium *spp. (Amaryllidaceae), *Hypericum *spp. (Hypericaceae), *Mentha *spp. (Lamiaceae), *Olea europea *L. (Oleaceae), and *Urtica dioica *L. (Urticaceae). Besides these few common species, however, there are many examples of medicinal plants being used in very distinct ways in different regions—even in areas sharing a similar flora, but a different cultural or linguistic heritage (Table 3). This point highlights the importance of documenting the TEK unique to diverse areas in Europe, as both unique preparations and medical applications of plants still commonly emerge.

### 3.3. Medical Ethnobotany in the Rest of Europe

In the other regions of Europe (i.e., in Central and Northern Europe), modern medical ethnobotanical studies are quite rare, due to the remarkable erosion of TK related to home-made plant-based remedies. In these countries, scholars have shifted their focus mainly to historical studies, using both scholarly and folkloric sources of information for their analyses (see, e.g., [217, 218] ). Indeed, the majority of the ethnobotanical literature on Europe is focused on Mediterranean regions, with the greatest number of publications based on ethnobotanical field studies conducted in Spain, Italy, and Turkey (Figure 1).

 On the other hand, CAM therapies of migrant communities in Northern Europe have presented an interesting topic of study, but most of these are dependent upon import of dried medicinal species from their cultural homeland (i.e., Africa, Asia, South America, Middle East, and etc.) and do not commonly incorporate the local flora [21, 41, 119, 120, 219–223]. The disappearance of autochthonous TK regarding CAM therapies in urban regions of northern Europe, where communities have less connectivity to the land and their natural resources, could be reflective of the future of southern Europe should the current trends in TK loss and erosion continue.

## 4. The Adaptive Nature of Traditional Pharmacopoeias

Local knowledge is not static; rather it is highly adaptive. It is open to adopt new species and techniques and to reject others. Transhumant shepherds, schoolteachers, monks, nuns, or migrants who return to small communities after periods away all help to introduce new plants and therapies. Moreover, the tragic events of wars and forced migrations also lead to the movement of both plants and sets of traditional knowledge from one cultural terrain to another. For example, remnants of ancient Albanian medicinal plant uses and names can still be found today amongst the Arbëreshë diaspora in Italy, who are descendants of Albanians that fled to southern Italy following the Ottoman Turk invasion of their homeland about 500 years ago (e.g., see [68, 209]).

People are highly likely to experiment with “new” remedies that had been previously used and praised by friends or relatives [150]. An excellent example of this phenomenon of knowledge transfer is that of *Eucalyptus globulus* Labill. (Myrtaceae), which was introduced in Cantabria, Spain, at the end on the nineteenth century and became very popular in a few decades and is nowadays an essential element of Cantabrian pharmacopoeia [190].

Many researchers have described a deep erosion of traditional medical knowledge following the deep social and economic changes of the past few decades (e.g., [68, 141, 224]). In many instances, herbal remedies are no longer used due to replacement with pharmaceuticals. Species like *Ruta chalepensis *L. (Rutaceae), which was very popular 50 years ago, are not commonly used nowadays. This is in spite of their common presence in people's front yards; common knowledge of their original function has been lost. The same has happened with other species such as *Lilium candidum *L. (Liliaceae)*, Syringa vulgaris* L. (Oleaceae),and *Iris germanica *L. (Iridaceae). In most cases, the memories of their medicinal applications have been lost and their roles have been restricted to environmental adornments [133].

Yet, on the other hand, researchers have observed an opposite trend with regards to a revitalization of traditional medical practices by youth and adult populations stemming from their concerns about the health risks of consuming industrial foods and pharmaceuticals [138]. In other words, an interest in pursuing a “natural” or healthier lifestyle as an alternative to the mainstream Western system has emerged and other alocthonous alternative herbs and medical systems such as acupuncture are being hybridized with local traditional health self-care practices and medicinal species. For example, commercial CAM products such as dietary supplements and nutraceuticals containing nonnative species like *Aloe vera *(L.) Burm. F (Xanthorrhoeaceae)*, Echinacea *spp. (Asteraceae), and *Panax ginseng* C. A. Mey (Araliaceae) are all becoming very popular [108, 225]. The type of consumers who typically use these products as CAM therapies do not commonly gather them, since they often lack both the access to the plants and the deep knowledge necessary for their collection and preparation [138]. This lack of TK of the local medicinal flora also restricts their use of CAM therapies to those that they can access through commercial markets, which rarely includes local species.

Despite this general trend of abandonment of local medicinal species, especially in urban populations, recent medicoethnobotanical and epidemiological studies have shown that botanicals do still play a critical role in rural healthcare. In particular, composites like *Chamaemelum nobile *(L.) All., *Matricaria recutita *L. or* Santolina chamaecyparissus *L. are still widely used throughout Spain (Table 2). In southern Italy, wild plants like *Cichorium intybus *L. (Asteraceae), *Leopoldia comosa *(L.) Parl. (Asparagaceae), and *Scolymus hispanicus* L. (Asteraceae) [209, 226] continue to make up a key part of the diet as functional health foods, whereas other plants like *Malva sylvestris *L. (Malvaceae), *Matricaria recutita *L. (Asteraceae), and *Marrubium vulgare *L. (Lamiaceae) are among the most important wild medicinals regularly gathered and used in household medicine [54, 68, 126]. 

Although there is an overall trend of decline of local medicinal plant use in urban areas, there are still examples of these practices, especially in southern Europe. For example, in Spain, city dwellers use medicinal plants such as *Aloysia citrodora *Paláu (Verbenaceae)*, Eucalyptus camaldulensis *Dehnh. (Myrtaceae)*, Matricaria recutita *L. (Asteraceae), *Mentha x piperita *L. (Lamiaceae)*, Santolina chamaecyparissus *L. (Asteraceae)*, Tilia platyphyllos *Scop. (Malvaceae),and* Thymus vulgaris* L. (Lamiaceae) [177, 227, 228]. These species are either gathered from the wild or bought. According to a survey conducted in Gandía (Valencia, Spain), 14% of the interviewees gathered them, and 11% obtained them from relatives or friends that had collected them [227]. In cities such as Barcelona, herbs are mainly bought in herbal shops or supermarkets [228]. Most of the herbs have a long tradition of use in the areas, and 43% of the participants in Barcelona answered that family tradition was the main reason for using them [228].

Some of these practices are even becoming more popular. As a result of tourism market that demands local authenticity, there are herbal infusions, such as *Jasonia glutinosa *DC. (Asteraceae) or *Sideritis hyssopifolia* L. (Lamiaceae) that are highly appreciated and which are even becoming symbols of local identity. They are offered in bars and restaurants and *S. hyssopifolia* is even being marketed in touristic areas such as Picos de Europa National Park [192].

Despite the fact that many of these species are well known in the scientific phytotherapy literature, there are highly valued plants that do not appear in modern phytotherapy treatises. For example, this is the case for both *Osmunda regalis *L. (Osmundaceae) and *Atractylis gummifera* L. (Asteraceae) used in Cantabria and Extremadura, respectively [193, 195]. Most people hide their use of these species from their doctor in order to avoid reprimand, since many Spanish allopathic practitioners lack adequate training in CAM and phytotherapy and tend to exhibit a sense of disdain towards traditional medicine, which is commonly seen as irrelevant or even harmful [143].

However, health policies cannot ignore the risks of an unsafe use of herbs. For example, in the case of *A. gummifera,* two recent poisonings were detected, one of them fatal, likely due to an accidental substitution of *Centaurea ornata* Willd. (Asteraceae) for *A. gummifera*. Health risks are increased by trends for self-medication and the consumers' perception that traditional herbal remedies are always safe and free of side effects [193]. It is, therefore, essential that health professionals adopt a culturally sensitive attitude towards traditional medicine and ask about the consumption of these remedies while taking the patient's medical history. 

## 5. Conclusions

Our review of the recent literature concerning medical ethnobotany in Europe highlights the dynamic nature of traditional knowledge concerning medicinal plants and traditional medical practices. While in some cases a resilience of local CAM practices has been observed, especially when ecotourism plays a role in creating a demand for authenticity of local products, this is not representative of most regions. In fact, alarmingly, many of the studies reviewed comment on the growing erosion of existing TK of folk medical practices that has accompanied acculturation processes and loss of linguistic diversity. In general, the younger generations are no longer able to identify the local flora that are useful as wild foods and medicines. In urban areas, those interested in continuing the incorporation of such products in their diet and lifestyle most often purchase them, or use other mainstream CAM products that are imported from other global sources. Likewise, migrant populations often import foreign medicinals to meet their health needs. 

Pluralistic and culturally appropriate approaches, which include “emic” views of newcomers' health seeking strategies, are increasingly considered crucial in our public health policies. In fact, these are often considered the only approaches that can build a genuine understanding of the holistic essence of health as a composite of physical, psychological, and social aspects of well-being. Understanding migrants' medical ethnobotanies can, therefore, offer a unique arena for fostering this aim, and for implementing the safe use of CAMs within the multicultural framework of diversity in the new Europe. 

Traditional knowledge of local health seeking strategies, including the use of local medicinal flora, can serve as a foundation for understanding small-scale uses of CAM natural products and allow us to assess the potential for the sustainable expansion of these practices into the larger European market as commercial CAMs. Medical ethnobotanical field studies can provide useful information to the allopathic medical community as they seek to reconcile existing and emerging CAM therapies with conventional biomedicine. This is of great importance not only for phytopharmacovigilance and managing risk of herb-drug interactions in mainstream patients that use CAM, but also for educating the medical community about ethnomedical systems and practices so that they can better serve growing migrant populations. Acculturation trends and economic shifts away from rural, agriculture-based local economies have contributed to a decline in knowledge of traditional health practices and TEK at large. All of these issues underline the critical importance of documenting the remaining traditional knowledge of local medicinal plants, especially in southern Europe, where it is still present and used in local health strategies.

## Figures and Tables

**Table 1:** Criteria considered for the inclusion or exclusion of studies in our analysis of medical field ethnobotany in Europe.

Inclusion criteria	Exclusion criteria
Medical ethnobotanical field studies	Meta-analyses were excluded if based on the data collected by others
Indexed in Scopus from 1992–2012	Works conducted on a single species or group of related species
Reports must provide precise details about the folk medicinal uses of plants	Field market surveys (unless the study involves folk-studies/TK of local or small-scale herb gathering and trade)
Works written in English (or have an English abstract)	Reports on large-scale trade of medicinal plants (i.e., commodities studies)

**Table 2:** Number and most important species (determined by highest consensus) in a selection of Iberian medical ethnobotany studies.

Study site	Number of medicinal plants	Reference	Most relevant species
Pallars (Catalonia, Spain)	437	[61, 64, 158]	*Thymus vulgaris* L. (Lamiaceae), *Sambucus nigra* L. (Adoxaceae)*, Juglans regia* L. (Juglandaceae), *Olea europaea* L. (Oleaceae), *Vitis vinifera* L. (Vitaceae)
Montseny (Catalonia, Spain)	351	[63]	*Sambucus nigra* L. (Adoxaceae), *Thymus vulgaris* L. (Lamiaceae), *Olea europaea* L. (Oleaceae), *Tilia platyphyllos* Scop. (Malvaceae), *Abies alba* Mill. (Pinaceae)
Cabo de Gata (Andalusia, Spain)	253	[111]	*Sideritis* sp.pl. (Lamiaceae), *Rosmarinus officinalis* L. (Lamiaceae)*, Ballota hirsuta* Benth. (Lamiaceae), *Marrubium vulgare* L. (Lamiaceae)*, Olea europaea* L. (Oleaceae)
W Granada province (Andalusia, Spain)	244	[81]	*Sideritis hirsuta* L. (Lamiaceae), *Rosmarinus officinalis* L. (Lamiaceae)*, Olea europaea* L. (Oleaceae), *Malva sylvestris* L. (Malvaceae), *Matricaria recutita* L. (Asteraceae)
Alta Vall del Ter (Catalonia, Spain)	220	[82]	*Arnica montana* L. (Asteraceae), *Hypericum perforatum* L. (Hypericaceae), *Thymus vulgaris* L. (Lamiaceae), *Sambucus nigra* L. (Adoxaceae), *Tilia platyphyllos* Scop. (Malvaceae)
Middle Navarra (Spain)	216	[79]	*Santolina chamaecyparissus* L. (Asteraceae)*, Jasonia glutinosa* DC. (Asteraceae), *Thymus vulgaris* L. (Lamiaceae), *Urtica dioica* L. (Urticaceae), *Chamaemelum nobile* (L.) All. (Asteraceae)
Arrabida (Setúbal, Portugal)	176	[84]	*Geranium purpureum *Vill. (Geraniaceae), *Rosmarinus officinalis* L. (Lamiaceae)*, Olea europaea* L. (Oleaceae), *Phlomis purpurea* L. (Lamiaceae), *Mentha pulegium* L. (Lamiaceae)
Northern Navarra (Spain)	174	[79]	*Chamaemelum nobile* (L.) All. (Asteraceae)*, Sambucus nigra* L. (Adoxaceae)*, Verbena officinalis* L. (Verbenaceae)*, Urtica dioica* L. (Urticaceae)*, Allium cepa* L. (Amaryllidaceae)
Montesinho (Tras-os-Montes, Portugal)	169	[159]	*T uberaria lignosa* (Sweet) Samp. (Cistaceae), *Olea europaea* L. (Oleaceae), *Linum usitatissimum* L. (Linaceae), *Juglans regia* L. (Juglandaceae), *Pterospartum tridentatum* (L.) Willk. (Fabaceae)
Campoo (Cantabria, Spain)	160	[160]	*Sambucus nigra* L. (Adoxaceae), *Rosmarinus officinalis* L. (Lamiaceae), *Urtica dioica* L. (Urticaceae), *Chamaemelum nobile* (L.) All. (Asteraceae)*, Equisetum* sp. pl. (Equisetaceae)
Vall del Tenes (Catalonia, Spain)	153	[108]	*Malva sylvestris* L. (Malvaceae), *Matricaria recutita* L. (Asteraceae), *Tilia platyphyllos* Scop. (Malvaceae), *Sambucus nigra* L. (Adoxaceae), *Salvia lavandulifolia* Vahl (Lamiaceae)
São Mamede (Portalegre, Portugal)	150	[62]	*Centaurium erythraea *Rafn (Gentianaceae), *Malva sylvestris* L. (Malvaceae),* Olea europaea* L. (Oleaceae)*, Pterospartum tridentatum* (L.) Willk. (Fabaceae), *Citrus sinensis* (L.) Osbeck (Rutaceae)
Serra da Açor (Central Portugal)	124	[161]	*Malva nicaeensis* All. (Malvaceae), *Sambucus nigra* L. (Adoxaceae), *Hypericum* sp. pl. (Hypericaceae), *Melissa officinalis* L. (Lamiaceae), *Sanguisorba verrucosa* (Link ex G. Don) Ces. (Rosaceae)
Piloña (Asturias, Spain)	107	[162]	*Chamaemelum nobile* (L.) All. (Asteraceae), *Ruta chalepensis* L. (Rutaceae), *Chelidonium majus* L. (Papaveraceae), *Origanum vulgare* L. (Lamiaceae), *Sideritis hyssopifolia* L. (Lamiaceae)
Sierra Mágina (Andalusia, Spain)	103	[163]	*Thymus zygis* L. (Lamiaceae), *Sideritis hirsuta* L. (Lamiaceae), *Ruta* sp. pl. (Rutaceae), *Olea europaea* L. (Oleaceae), *Eucalyptus camaldulensis* Dehnh. (Myrtaceae)
Riverside Navarra (Spain)	90	[80]	*Santolina chamaecyparissus* L. (Asteraceae)*, Thymus vulgaris* L. (Lamiaceae), *Rosmarinus officinalis* L. (Lamiaceae)*, Urtica dioica *L.* (Urticaceae), Malva sylvestris *L.* (Malvaceae) *
Segarra (Catalonia, Spain)	92	[110]	*Thymus vulgaris* L. (Lamiaceae), *Malva sylvestris* L. (Malvaceae), *Rosmarinus officinalis* L. (Lamiaceae), *Papaver rhoeas* L. (Papaveraceae), *Salvia lavandulifolia* Vahl (Lamiaceae)
Chaves, Montalegre (Tras-os-Montes, Portugal)	88	[34]	*Salvia officinalis* L. (Lamiaceae), *Plantago major* L. (Plantaginaceae), *Thymus pulegioides* L. (Lamiaceae), *Hypericum perforatum* L. (Hypericaceae)
Gorbeialdea (Basque Country, Spain)	82	[164]	*Urtica dioica* L. (Urticaceae), *Chamaemelum nobile* (L.) All. (Asteraceae), *Plantago* sp. pl. (Plantaginaceae), *Verbena officinalis* L. (Verbenaceae), *Chelidonium majus* L. (Papaveraceae)

**Table 3:** Number and most important species (determined by high consensus) in a selection of south European medical ethnobotany studies.

Study site	Number of medicinal plants	Reference	Most relevant species
Inland Marches, Italy	70	[208]	*Allium cepa* L. (Amaryllidaceae), *Avena sativa* L. (Poaceae), *Balsamita major* (L.) Desf. (Asteraceae), *Calendula officinalis* L. (Asteraceae), *Castanea sativa* L. (Fagaceae), *Centaurea cyanus* L. (Asteraceae), *Daucus carota* L. (Apiaceae), *Hedera helix* L. (Araliaceae), *Hypericum perforatum* L. (Hypericaceae), *Juglans regia* L. (Juglandaceae), *Lavandula angustifolia* Mill. (Lamiaceae), *Malva sylvestris* L. (Malvaceae), *Matricaria recutita* L. (Asteraceae), *Ocimum basilicum* L. (Lamiaceae), *Papaver rhoeas* L. (Papaveraceae), *Prunus dulcis* (Mill.) D.A. Webb (Rosaceae), *Rosa canina* L. (Rosaceae), *Rosmarinus officinalis* L. (Lamiaceae), *Rubus fruticosus* L. (Rosaceae), *Salvia officinalis* L. (Lamiaceae), *Sambucus nigra* L. (Adoxaceae), *Solanum tuberosum* L. (Solanaceae), *Spartium junceum* L. (Fabaceae), *Urtica dioica* L. (Urticaceae)
Dolomiti Lucane (Basilicata), Italy	103	[126]	*Allium cepa* L. (Amaryllidaceae), *Cynodon dactylon* (L.) Pers. (Poaceae), *Euphorbia cyparissias* L. (Euphorbiaceae), *Hordeum vulgare* L. (Poaceae), *Hypericum hircinum* L. (Hypericaceae), *Laurus nobilis* L. (Lauraceae), *Matricaria recutita* L. (Asteraceae), *Malva sylvestris* L. (Malvaceae), *Malus domestica* Borkh. (Rosaceae), *Vitis vinifera* L. (Vitaceae)
Arbëreshë (ethnic Albanians in N. Basilicata), Italy	120	[68, 209]	*Allium cepa* L. (Amaryllidaceae), *Allium sativum* L. (Amaryllidaceae), *Agropyron repens* L. (Poaceae), *Arundo donax* L. (Poaceae), *Borago officinalis* L. (Boraginaceae), *Cichorium intybus* L. (Asteraceae), *Ficus carica* L. (Moraceae), *Hordeum vulgare* L. (Poaceae), *Malus domestica* Borkh. (Rosaceae), *Malva sylvestris* L. (Malvaceae), *Marrubium vulgare* L. (Lamiaceae), *Matricaria recutita* L. (Asteraceae), *Olea europea* L. (Oleaceae), *Vitis vinifera* L. (Vitaceae)
Gollak region, Kosovo	92	[214]	*Allium cepa* L. (Amaryllidaceae), *Cornus mas* L. (Cornaceae), *Crataegus monogyna* Jacq. (Rosaceae), *Fragaria vesca* L. (Rosaceae), *Hypericum perforatum* L. (Hypericaceae), *Malus sylvestris* Mill. (Rosaceae), *Matricaria chamomilla* L. (Asteraceae), *Origanum vulgare* L. (Lamiaceae), *Plantago major* L. (Plantaginaceae), *Prunus cerasus* L. (Rosaceae), *Prunus persica* (L.) Batsch (Rosaceae), *Rubus idaeus* L. (Rosaceae), *Urtica dioica* L. (Urticaceae)
Prokletije Mountains (Montenegro)	94	[18]	*Achillea millefolium* L. (Asteraceae), *Hypericum perforatum* L. (Hypericaceae), *Rosa canina* L. (Rosaceae), *Sambucus nigra* L. (Adoxaceae), *Thymus serpyllum* L. (Lamiaceae), *Urtica dioica* L. (Urticaceae), *Vaccinium myrtillus* L. (Ericacaeae)
Pešter Plateau, Sandžak, SW Serbia	62	[213]	*Chenopodium bonus-henricus* L. (Amaranthaceae), *Gentiana lutea* L. (Gentianaceae), *Origanum vulgare* L. (Lamiaceae), *Hypericum* spp. (Hypericaceae), *Rosa canina* L. (Rosaceae), *Urtica dioica* L. (Urticaceae)
Sivrice (Elaziğ), Turkey	81	[23]	*Thymus haussknechtii *Velen, (Lamiaceae), *Mentha spicata* L. (Lamiaceae), *Malva neglecta* Wallr. (Lamiaceae), *Rosa canina* L. (Rosaceae), *Hypericum perforatum* L. (Hypericaceae), *Rheum ribes* L. (Polygonaceae), *Rubus discolor* Weihe & Nees (Rosaceae), *Portulaca oleracea* L. (Portulacaceae), *Urtica dioica* L. (Urticaceae)
Maden (Elaziğ), Turkey	88	[15]	*Mentha spicata* L. subsp. *spicata* (Lamiaceae), *Rosa canina* L. (Rosaceae), *Urtica dioica* L. (Urticaceae), *Anthemis wiedemanniana* Fisch. and C.A. Mey. (Asteraceae), *Bunium paucifolium* DC. var. *brevipes* (Freyn & Sint.) Hedge & Lam. (Apiaceae), *Tchihatchewia isatidea* Boiss. (Brassicaceae), *Thymus haussknechtii* Velen. (Lamiaceae)
